# Accuracy of computer-guided implant surgery in partially edentulous patients: a prospective observational study

**DOI:** 10.1186/s40729-024-00552-z

**Published:** 2024-07-16

**Authors:** Emile Chrabieh, Christine Hanna, Stephanie Mrad, Stephanie Rameh, Joseph Bassil, Joseph Zaarour

**Affiliations:** 1 Department of Oral Surgery, Saint Joseph University of Beirut, Beirut, Lebanon; 2 Department of Prosthetic and Restorative Dentistry, Saint Joseph University of Beirut, Beirut, Lebanon; 3 Division of Prosthodontics, Department of Restorative Sciences, School of Dentistry, University of Minnesota, Minneapolis, USA; 4 Faculty of dental medicine, Campus of Medical Sciences, Saint Joseph University of Beirut, Damascus Road, Beirut, 1104 2020 Lebanon

**Keywords:** Accuracy, Dental implant, CAD-CAM, Guided surgery, Partially edentulous, Surgical guide, Surgical template

## Abstract

**Purpose:**

This study aims to evaluate the amount of distortion using computer-guided implant surgery with 3D printed surgical guides in limited edentulous spaces.

**Materials and methods:**

25 bone level self-tapping implants (Straumann® BL and BLT) were randomly inserted in either distal or intercalary posterior mandibular edentulism using a fully digital protocol and 3D printed surgical guides. Amount of inaccuracy was evaluated after superimposing the 3 coordinates of virtually planned and final implant images, which were obtained using intra-oral scans and scan bodies. Four evaluation parameters were considered: origo-displacement, error depth, apical displacement and angle between the planned and the placed implant.

**Results:**

The average of distortion was 0.71 mm for the origo-displacement, 0.36 mm for the error depth, 0.52 mm for the horizontal displacement and 3.34º for the error angle.

**Conclusion:**

The major reason of exclusion was CBCT artifacts. Results of this study were aligned with the results of previous studies concerning partially edentulous spaces. CAD/CAM manufacturing process did not result in significant distortion whilst the biggest part of distortions originated from the surgical process. The learning curve in computer-guided implant surgery presented an important source of inaccuracy.

**Supplementary Information:**

The online version contains supplementary material available at 10.1186/s40729-024-00552-z.

## Trial registry

This trial is registered to the ClinicalTrials.gov Protocol Registration.

- Name of the registry: ClinicalTrials.gov Protocol Registration.
- Trial registration number: NCT06277076.
- Date of registration: February 17, 2024 Retrospectively registered.
- URL of trial registry record: https://register.clinicaltrials.gov/NCT06277076.

**Supplementary Information:**

The online version contains supplementary material available at 10.1186/s40729-024-00552-z.

## Introduction

Static Implant guided surgery (SIGS) or Computerized Implant Guided Surgery (CIGS) is nowadays commonly used in our dental practice. In this digital workflow, implant positioning is planned pre-operatively based on cone beam computed tomography (CBCT) and optical scan of the patient, and the surgical procedure is then performed accordingly using templates [1]. Besides optimal 3D implant positioning, guided surgery has several advantages such as better prosthetically-driven implant placement, respect of the neighboring anatomy, and reduced treatment duration. Furthermore, it presents a precious communication tool to discuss treatment options with prosthodontists, lab technicians, as well as patients. All these inputs considerably enhance the esthetic and functional outcomes of implant treatments [2–4].

Accuracy and precision of implant placement are crucial in cases of adjacent anatomical structures, limited crestal volume, or planned immediate loading protocol [5]. Static guided implant surgery showed better accuracy than both free-hand [6–8] and dynamic guided implant surgery (navigation surgery) [9]. However, static guided implant surgery consists of several steps, and each one could present various sources of distortions. The cumulative amount of these distortions constitutes the final amount in terms of inaccuracy.

Among these sources of distortion is the superimposition of CBCT images and intra-oral surface scan during planning, especially in cases of artifacts. Also, the gap between sleeve and spoon, gap between drills and spoon, length of the drills, drilling movement as well as skills of the surgeon are sources of inaccuracy in computer-guided protocols [10, 11]. 

Moreover, the concerned length of edentulism is highly related to the amount of distortion. While most articles on guided-surgery accuracy combine the results of all types of edentulism, a recent systematic review highlights the difference in results between fully and partially edentulous cases. Thus, the amount of deviation varies based on the type of edentulism. The larger the edentulous space, the higher is the inaccuracy of guided implant positioning [12]. This is in relation with the type of tissue supporting the surgical guide. For instance, mucosa and tooth-supported guides are far better than bone-supported ones in terms of stability and accuracy [13]. 

Accordingly, several techniques, such as fixation pins and temporary implants, are proposed to stabilize the surgical guide and reduce distortion values. Also, addition of supportive reinforcement bars within the resin material of surgical guides is used to avoid bending and limit deviations [14]. 

The present study focuses on partially edentulous jaws, specifically distal-shortened mandible and bounded spaces of two to three teeth. The aim is to evaluate the accuracy and precision of fully guided implant surgery in partially edentulous mandibular spaces.

## Materials and methods

### Population

Patients presenting at Saint Joseph University of Beirut dental clinics with a distal-shortened mandible or distal intercalary space of two to three missing premolars or molars (class I, II or VI according to Kennedy’s classification on edentulous spaces) and requiring implant-supported reconstructions were selected to participate in this study.

For each case two implants were placed with a fully guided procedure followed by the insertion of two to three splinted temporary resin crowns that were virtually designed and prepared. Temporary crowns were loaded either immediately or conventionally (after 2 months) depending on primary stability.

Thirteen patients were included in this study with twenty-five implants eligible for accuracy evaluation. Deviation of final implant position compared to virtually planned implant position was assessed after superimposing the two scanned 3D images.

#### Inclusion criteria

- Age over 18 years old.
- Acceptable oral hygiene with no signs of active periodontal disease or local infection.
- ASA 1 and 2.
- Non or Light smoking (< 10 cigarettes per day).
- Acceptable intermaxillary space (44 mm minimum).
- Minimal crestal width of 5.5 mm.
- Minimal residual bone height of 10 mm above the alveolar nerve canal.

#### Exclusion criteria

- Head and neck irradiation.
- Bone grafted sites.
- Severe bruxism.
- CBCT with exaggerated artifacts.
- Limited intermaxillary space.

An Institutional Review Board (IRB) approval was given by the Committee for the Protection of Human Subjects (CPHS) of the Saint Joseph University of Beirut-Lebanon, and an informed consent was signed by all patients.

### Pre-operative planning

A pre-operative CBCT was taken with an open mouth of 2 cm to prevent any overlap or artifact from the opposite arch. DICOM files (Digital Imaging and Communications in Medicine) derived from the CBCT were imported into Implant Studio (3Shape® Copenhagen, Denmark) planning software.

**Fig. 1:**
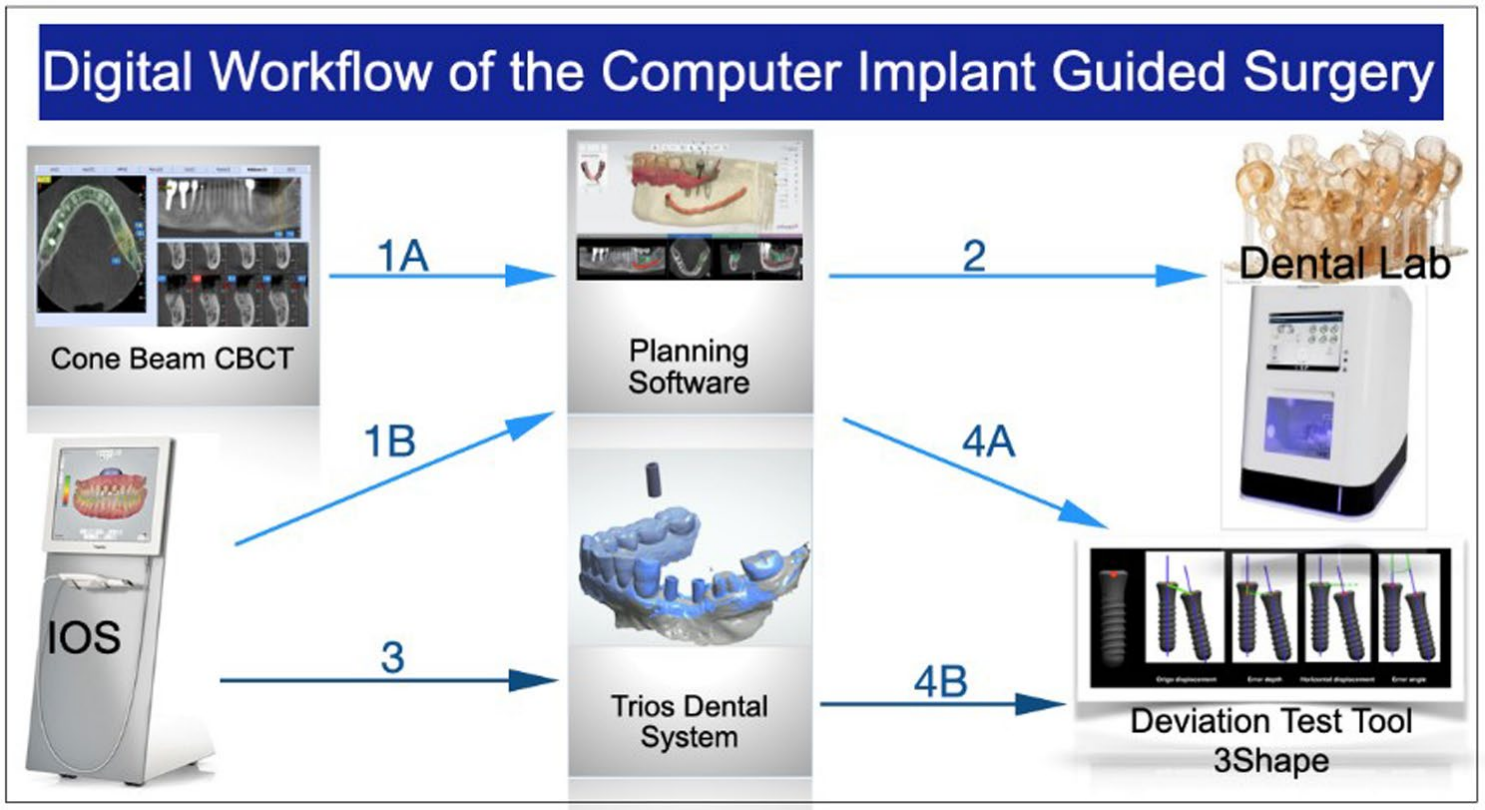
The 4 stages of the digital workflow leading to deviation assessment. Stage 1 and 2 are pre-implant placement; Stage 3 and 4 are post-implant placement. 1 A- DICOM/DCM File exported from CBCT to the planning software for super-imposition; 1B- 3OXZ file exported from the intra-oral scanner to Implant Studio software for superimposition and implant planning; 2- STL file used to print the surgical guide and 3OXZ file used to design and mill the crowns in the dental Lab; 3- Implant scanning and 3OXZ file export to Trios Dental System for flag matching with the scan-body; 4-The Information of the planned implant (4 A) and the placed implant (4B) with coordinates embedded are exported as DCM files to the deviation test tool. *(3OXZ*: 3Shape Order Exchange Zip File; *DICOM/DCM*: Digital Imaging and Communications in Medicine; *STL*: Standard Tessellation Language)

An optical surface scan was performed using TRIOS (3Shape® Copenhagen, Denmark) optical scan. The resulting 3oxz file (optical 3D image) was exported to the Implant Studio software as well (stage 1 A and 1B Fig. 1).

The two 3D images were subsequently superimposed and implant placement was planned in a prosthetically-driven manner. A 2 mm security distance from the alveolar nerve, that was set by the 6th ITI consensus conference in 2018 (6), was respected in this study.

In case of distal edentulous space, a lateral fixation pin was planned to strengthen the guide, prevent its distal bending, and achieve multiple tissue support (mucosal, dental and pin support). After implant planning and guide design (see stage 2 Fig. 1), an STL file (Stereo Lithography interface format) was obtained for guide printing, a 3oxz file for temporary resin crowns fabrication, and a DICOM file for future superimposition with the final implants position (stage 4 A Fig. 1).

### Surgical guide and crown manufacturing

**Fig. 2:**
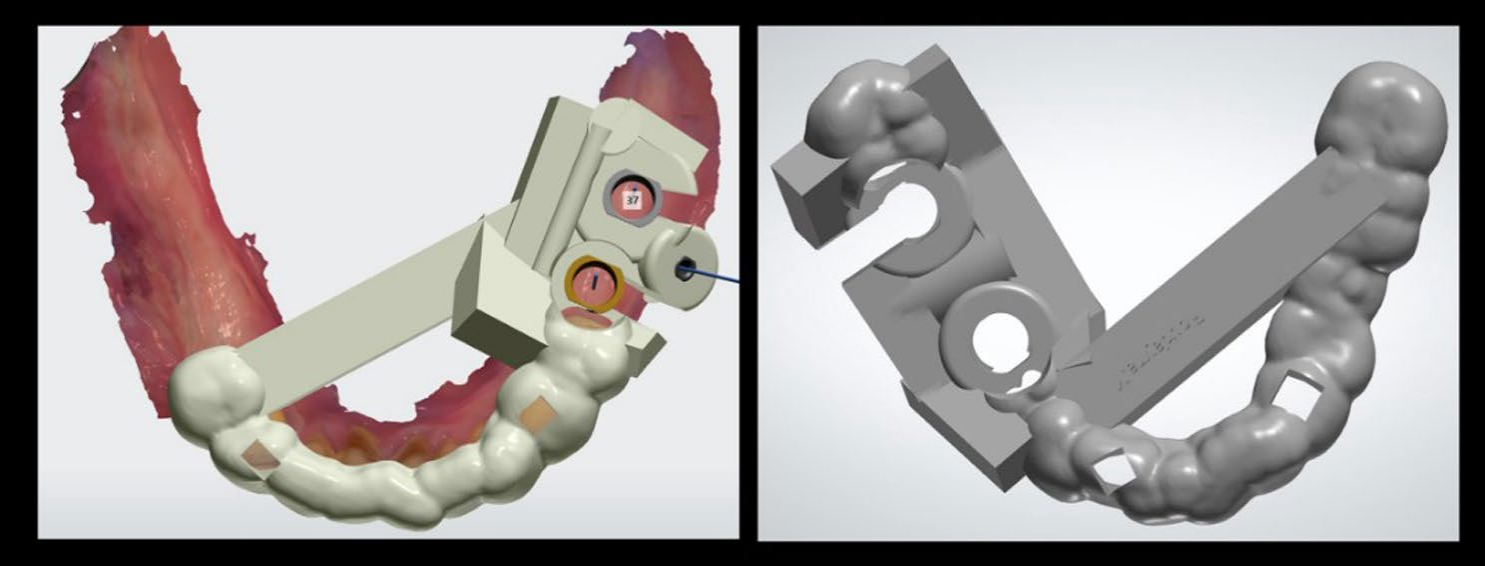
Surgical guide with reinforcement bars, fixation pin for distal edentulous spaces, lateral windows for posterior sites and occlusal windows for seating control

STL files were used to print the resin surgical guide with a 3D printer - Envisiontec (Envisiontec Vida HD, Envisiontec Inc, USA). Lateral windows for seating check, additional bars for reinforcement and metal rods inserted into the resin for higher rigidity were designed (Fig. 2).

For immediate loading protocols, virtual splinted crowns were designed using a Dental System DS Software (3Shape, Copenhagen, Denmark) and produced by a milling machine (Amann Girrbach AG, Koblach, Austria).

**Fig. 3:**
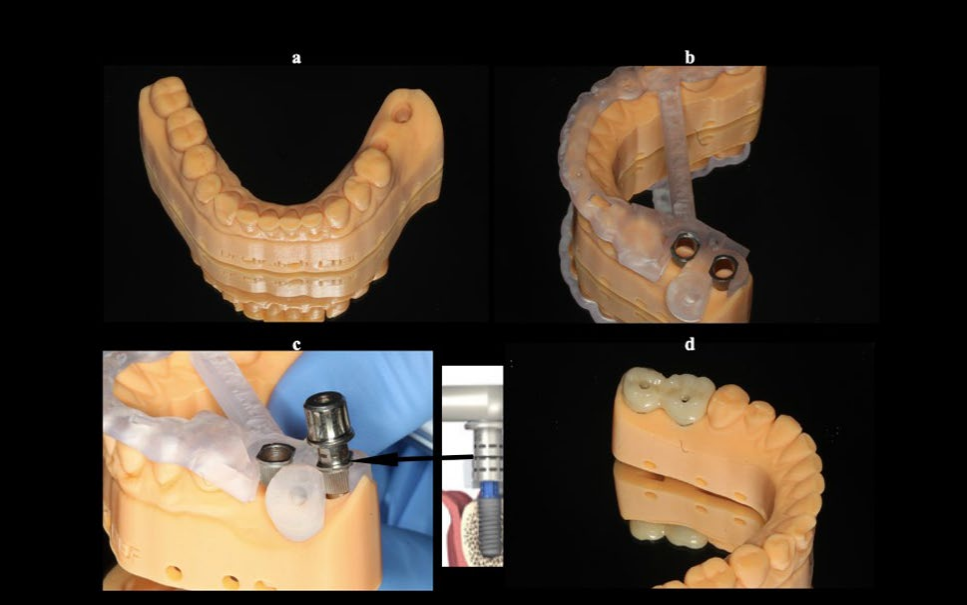
Simulation proving the accuracy of the pre-operative steps related to the digital workflow: a- Printed cast with preparation for implant digital analog. b- Control of the surgical guide seating. c- Simulation of free-hand implant insertion by checking the laser-mark of the implant holder. d- Positioning the screw-retained splinted crowns to validate the accuracy of their seating once again

To basically evaluate the accuracy of CAD/CAM processes, a mandibular cast was printed with replicas of planned implants inserted into this model, and three tests were effectuated (Fig. 3). Firstly, the surgical guide fit was checked by seating the later over the printed model and appraising its stability. Secondly, implant placement procedure was tested performing a simulation and evaluating if the laser mark of implant holder corresponds to the planned level of offset. Thirdly, the crown fit was assessed on the corresponding screw-retained Variobase abutments from Straumann. These tests proved the accuracy of pre-operative steps related to the digital workflow. Thus, CAD/CAM protocol was free from any source of distortion.

In cases of reduced mouth opening, a lateral window was created in the sleeve and guide to prevent any pressure during drilling procedures.

### Guided implant surgery protocol

Only one experienced and skilled investigator was in charge to perform all the surgeries in respect with the consistency of the protocol.

**Fig. 4:**
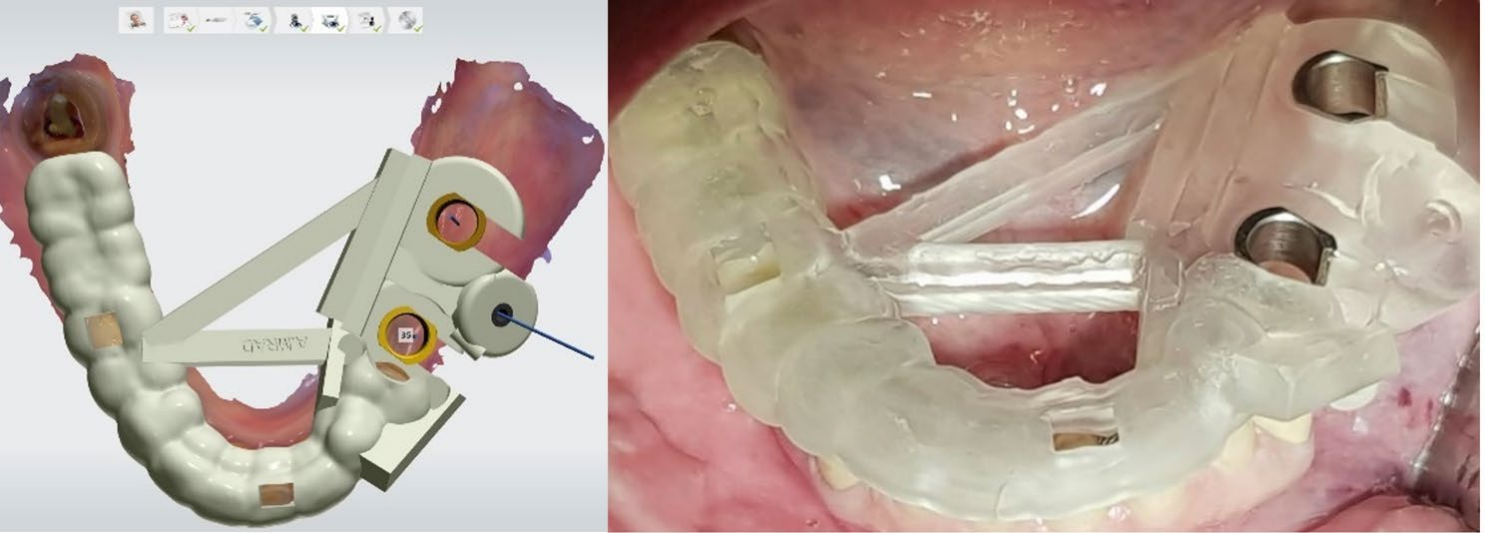
Design of the surgical guide with fixation pin and fitting of the printed guide intra-orally during surgery

After local anesthesia, the surgical guide was seated, 1.25 mm fixation pins (Straumann) were placed, stability of the surgical guide was checked, and special attention for rocking or loose fit of the guide was accorded. Surgical guides were tooth-mucosa supported and were stabilized with fixation pins (Fig. 4). Depending on the thickness of the tissue and the amount of keratinized tissue around the implantation area, the investigators decided to either choose a flapless or open-flap procedure.

The followed drilling protocol was according to the implant studio planning, using adequate guided surgical cassette, drills with stoppers, and metallic drill handles. After implant site preparation, implants were inserted in a fully guided manner through the sleeve using a guided surgery implant holder with corresponding off-set laser marks. Bone level (BL) and bone level tapered (BLT) Roxolid implants (Straumann AG, Basel, Switzerland) with a diameter of either 3.3–4.1 mm and length of 8–10 mm were chosen.

After the surgery, patients were asked to take the following medications for seven days: Diclofenac as NSAID twice daily, Paracetamol 1 g if needed and mouthwash with Chlorhexidine Digluconate 0.12% three times per day.

### Measured parameters

#### 1. Primary stability assessment

_ At the last rotation during implant insertion, a customized adaptor for the digital ratchet of the insertion torque (IT) device (DTA, by studio AIP Srl, Italy) was used as an implant holder and the values were recorded with a transducer connected to a computer via Bluetooth wireless. A graph displayed the variation of IT with each rotation on DT1 2.2 software, and the highest value was considered the maximum insertion torque in Ncm.

_ Implant stability quotient ISQ was also recorded following the resonance frequency analysis RFA using the Osstell AB ISQ device (Ostell ISQ; Integration Diagnostics AB, Göteborg, Sweden).

The ISQ records were only scored to follow with time the evolution of the implant stability, since it is considered as an assessment which can provide prospective monitoring and shows fluctuations in stiffness of the implant interface as bone matures from primary to secondary contact [15].

In the current study the ISQ was only a variable used to monitor the behavior of the secondary stability after immediate loading.

**Fig. 5:**
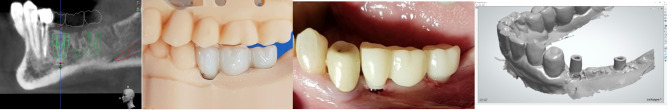
Planning and clinical view of an Immediate loading of implants with high primary stability over a screw retained bridge and a surface scan with digital scan bodies of the placed implants

_ When primary stability of the two implants reached 30 Ncm and above, an immediate loading protocol was applied by inserting two splinted, prefabricated and milled screw-retained crowns on top of Variobase abutments for bridge (Straumann AG, Basel, Switzerland). (Fig. 5)

#### 2. Assessment of the deviations

**Fig. 6:**
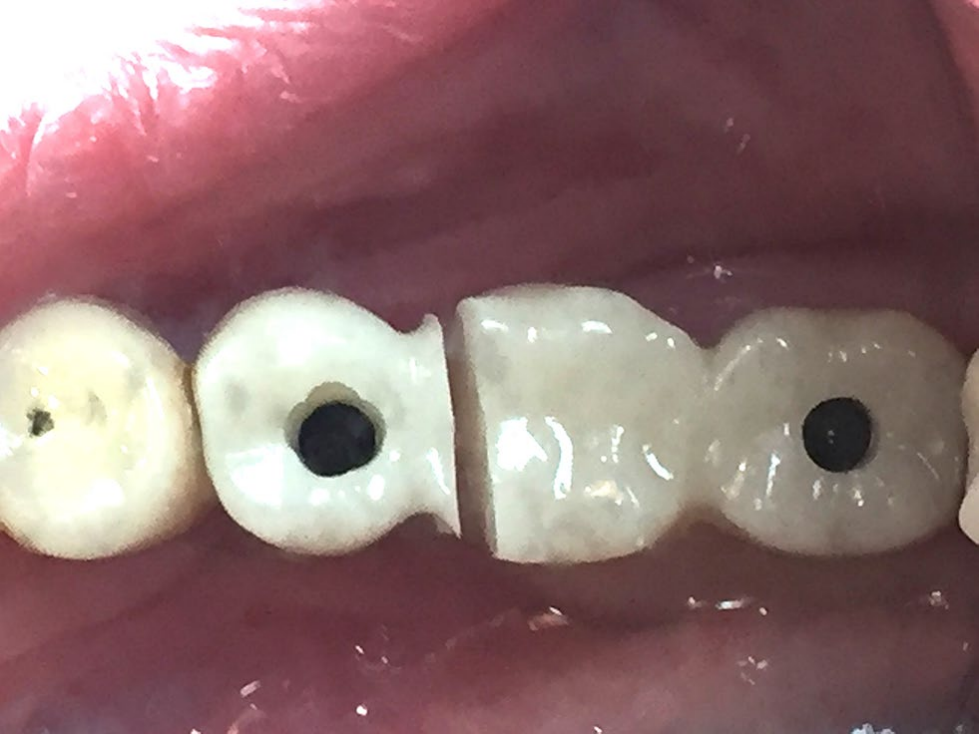
Temporary restoration cut with a disc to repair it for a passive fit

A preliminary clinical visual check of the distortions was observed at the moment of insertion of the pre-milled splinted crowns. For adjustment of misfitted temporary restorations due to inaccuracy of implant placement, a relining or reparation with resin material was performed to ensure a passive fit (Fig. 6).

**Fig. 7:**
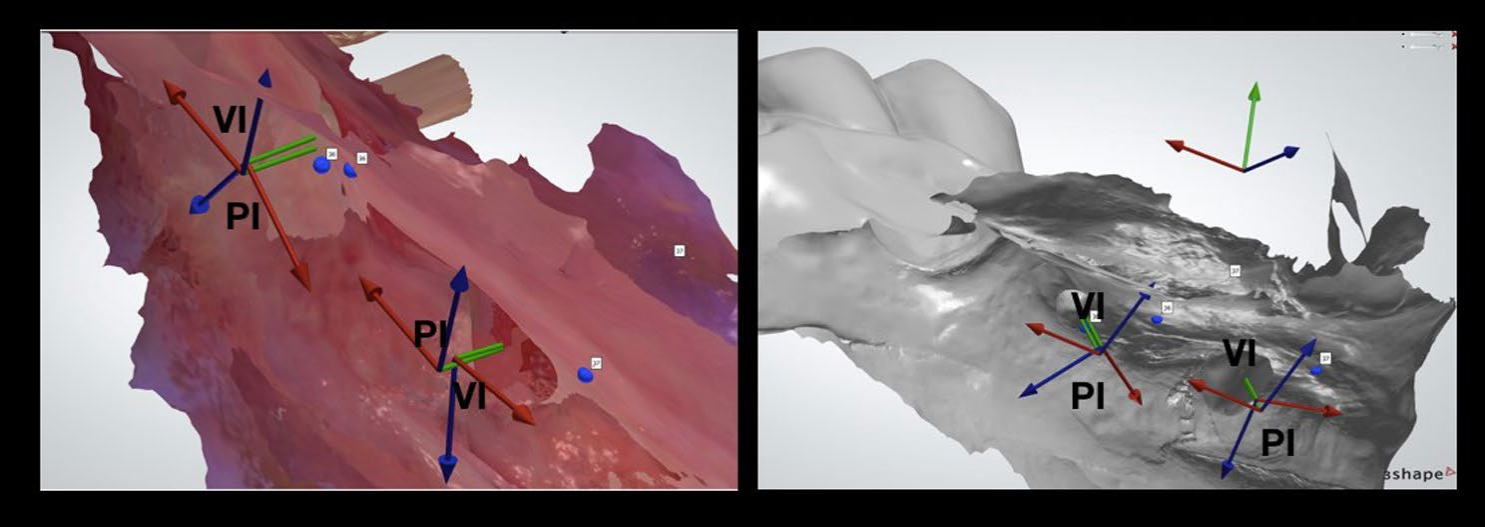
3D Viewer software was used to assess the amount of deviation: the 3 coordinate arrows of the Virtual (VI) and the placed (PI) implants were superimposed. Also, the accuracy of the mesial implant (neighboring to natural tooth) was compared to the distal implant

The real assessment of the deviations was performed only at eight weeks after implant placement. Intra-oral surface scans of the final implants’ positions were registered using a digital scan body. Resulting 3oxz output files were exported to the Dental system DS software of 3Shape to generate a DCM file of the placed implant. Afterwards, the deviations between virtual implant VI and placed implant PI positions were calculated with a “comparison tool” developed by 3Shape company for research purposes (Fig. 7; stage 4 Fig. 1).

**Fig. 8:**
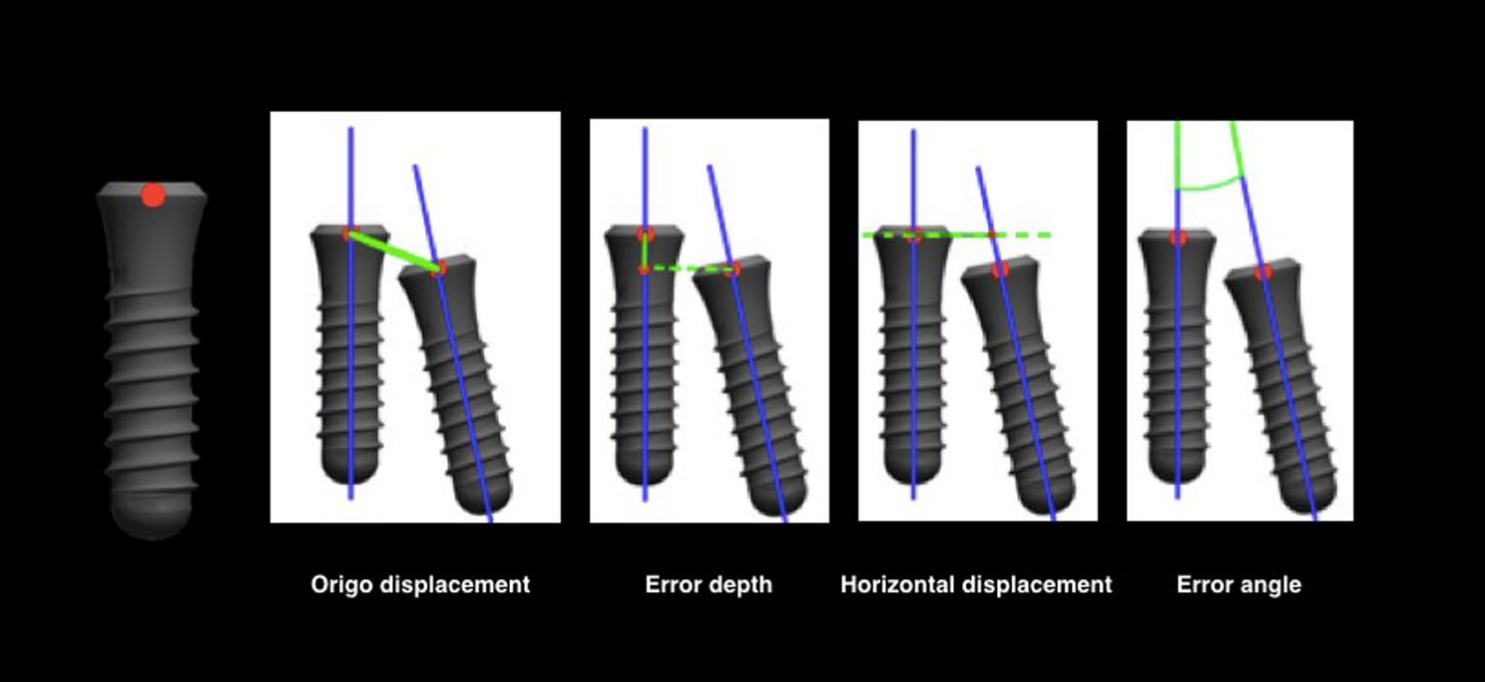
Deviation parameters obtained with the implant position comparison tool from 3Shape

Four different measures were provided to calculate the amount of distortion in millimeter (mm) or in degree: Origo Displacement OD (mm), Horizontal Displacement HD (mm), Error Depth ED (mm) and Error Angle EA (degree) (Fig. 8).

## Results

Mean deviation of parameters between VI and PI positions for 25 implants was: 0.71 mm for Origo-Displacement, 0.36 mm for Error Depth, 0.52 mm for Horizontal Displacement and 3.34º for Error Angle (Table 1). Minimal and maximal deviation values of the four parameters are presented in Table 1.

**Table 1:** Deviations of the four parameters in all samples

In all the sample	*N*	Minimum	Maximum	Mean	Std. deviation	95% Confidence interval for mean	-*p*-value comparison with 0
Origo displacement	25	0.13	1.25	0.71	0.28	0.59–0.82	< 0.001
Error Depth	25	0.01	0.80	0.36	0.28	0.25–0.48	< 0.001
Horizontal displacement	25	0.12	1.25	0.52	0.27	0.41–0.63	< 0.001
Error Angle	25	1.38	8.04	3.34	2.12	2.47–4.21	< 0.001

PI showed significantly better accuracy in ED compared to HD. Minimal ED was 0.01 mm and maximal ED was 0.8 mm, while minimal HD was 0.12 mm and maximal HD was 1.25 mm.

**Fig. 9:**
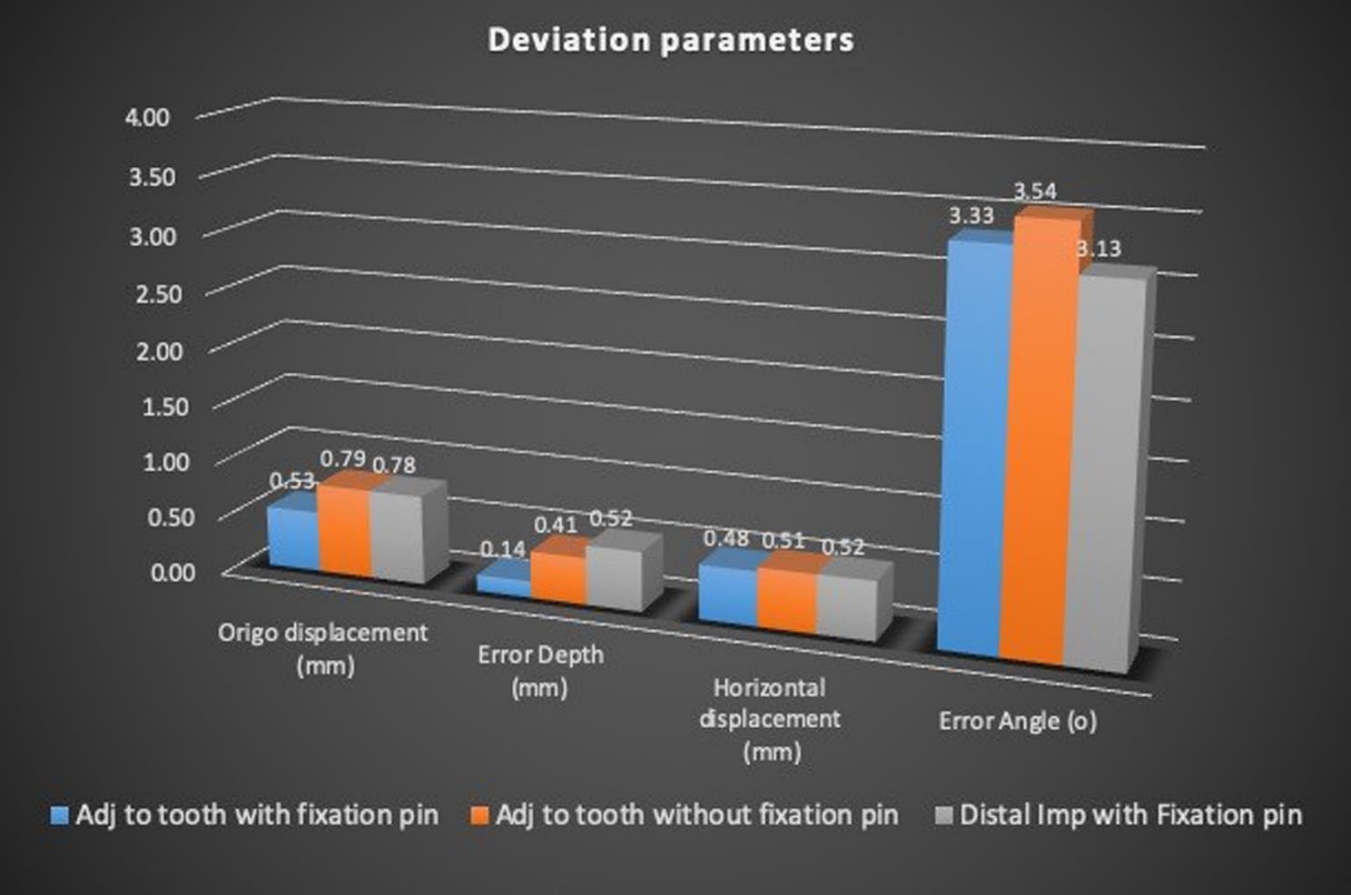
Deviation parameters in different site situations

Difference in accuracy between intercalated implants with or without fixation pins and distally placed implants with fixation pins was not statistically significant. Results are divided into groups depending on the type of tissue support (Fig. 9).

Also, difference between the two different implant macro-designs in terms of accuracy was not statistically significant. BL and BLT Straumann implants showed respectively the following values of distortions: 0.67 and 0.74 mm for OD, 0.35 and 0.44 mm for ED, 0.49 and 0.56 mm for HD, and 3.36º and 3.31º for EA (Table 2).

**Table 2:** Comparison of the deviations between the two types of implants BL and BLT

		*N*	Minimum	Maximum	Mean	Std. deviation	95% Confidence interval for mean	*p*-value comparison with 0	*p*-value comparisons between groups
Origo displacement	BL	13	0.13	1.12	0.67	0.29	0.50–0.85	< 0.001	0.550
	BLT	12	0.33	1.25	0.74	0.27	0.57–0.92	< 0.001	
Error Depth	BL	13	0.01	0.80	0.35	0.27	0.19–0.51	0.001	0.809
	BLT	12	0.01	0.80	0.44	0.31	0.18–0.57	0.001	
Horizontal displacement	BL	13	0.12	1.02	0.49	0.26	0.33–0.64	< 0.001	0.508
	BLT	12	0.24	1.25	0.56	0.29	0.38–0.74	< 0.001	
Error Angle	BL	13	1.38	8.04	3.36	2.27	1.99–4.74	< 0.001	0.951
	BLT	12	1.38	8.04	3.31	2.03	2.02–4.60	< 0.001	

Implant length showed difference in accuracy between the two implants used in the study. Mean values of 8 mm and 10 mm implants were respectively 0.87 and 0.63 mm for OD, 0.39 and 0.34 mm for ED, 0.67 and 0.46 mm for HD, and 3.50º and 3.18º for EA. Results showed better accuracy for longer implants, however they were not statistically significant. (Table 3).

**Table 3:** Comparison of the deviations between the two implant lengths

		*N*	Minimum	Maximum	Mean	Std. Deviation	95% Confidence Interval for Mean	*p*-value comparison with 0	*p*-value comparisons between groups
Origo displacement	8 mm	7	0.40	1.25	0.87	0.30	0.59–10.15	< 0.001	0.064
	10 mm	18	0.13	1.10	0.63	0.25	0.52–0.77	< 0.001	
Error Depth	8 mm	7	0.01	0.69	0.39	0.30	0.11–0.67	0.014	0.763
	10 mm	18	0.01	0.80	0.34	0.28	0.21–0.49	< 0.001	
Horizontal displacement	8 mm	7	0.24	1.25	0.67	0.40	0.30 -10.03	0.004	0.096
	10 mm	18	0.12	0.78	0.46	0.19	0.37–0.56	< 0.001	
Error Angle	8 mm	7	1.38	8.04	3.50	2.16	1.50–5.50	0.005	0.815
	10 mm	18	1.38	8.04	3.18	2.16	2.20–4.35	< 0.001	

## Discussion

Guided implant surgery is comprised of several steps and each one could be a source of inaccuracy [12]. Firstly, radiographic artifacts may embed the superimposition of CBCT and digital scan surfaces as per many cases that were excluded from this study. Different CBCT sources, software, and segmentation techniques were proposed to reduce these artifacts but could not completely resolve this limitation [16]. 

Secondly, the process of CAM or digital manufacturing of the surgical guides and temporary crowns. In the current study, 3D printing the models were printed and the fit of surgical guides and milled crowns was tested. These control tests showed great accuracy; thus, deviations mainly resulted from the superimposition or the surgical phase.

Another source of deviation is limited inter-arch space which may prevent free insertion of drills into the sleeves and cause bending of the surgical guide. This issue could not be completely resolved even after creating lateral windows in the sleeves. This possible source of distortion was excluded from this study.

Several studies proved that a certain void exist between both sleeve and drill handle, and between drill handle and drill, which is responsible for a certain amount of deviation [17]. In all cases of this study, adequate guide retention and adaptation was ensured to avoid distortions and bending, sleeves were embedded into the resin to guarantee their rigid stabilization, and bars were added to reinforce the guide.

Moreover, Herekar et al. related implant macro-design to CIGS precision. Cylindrical implants were more likely to interfere with the cortical wall or be pushed away from it while tapered implants with narrow apexes were supposed to penetrate the cortical wall or stay away from it [12]. This is not in accordance with the results of this study where the difference between BL and BLT implants in terms of accuracy was not statistically significant.

Concerning implant length, 8 mm implants showed more distortion than the 10 mm implants in the current study. Similarly, Derksen et al. evaluated 145 implants and found long implants of 12 mm with no cortical interference to have significantly better accuracy in angular deviations than shorter implants [13]. Conversely, Vercruyssen et al. did not find any influence of implant length on CIGS accuracy [18]. While several other studies found that long implants have higher deviations at the apex. Accordingly, decreasing the drilling distance below the guided sleeve, by using shorter sleeve heights or shorter implants, could significantly increase the accuracy of CIGS [19–21].

Several studies evaluated the influence of tissue support on the accuracy of CIGS. A recent systematic review proved that mucosal-supported guides show significant reduction in angular deviation (*P* = 0.02), point-of-entry deflection (*P* = 0.002), and tip deviation (*P* = 0.04) when compared to bone guides. Whereas mucosa-supported and teeth-supported guides had no statistically significant difference for any of the outcome measures [22]. For these types of guides, Verhamme et al. recommended the use of fixation screws to decrease the bucco-lingual deviation. Fixation screws were used to stabilize all surgical guides in the present study, which explains the reduced deviation of OD and HD values [21]. 

On the other hand, this study was limited to edentulous spaces of two adjacent teeth without considering fully edentulous jaws, which explains the low level of cumulative distortions. A meta-analysis showed that partially edentulous cases have a significantly higher accuracy at entry point and at the apex when compared to fully edentulous cases [12]. Schnutenhaus in a study on partially edentulous spaces did not find a significant difference between implants placed in distal edentulous spaces and those in single tooth gaps [23]. These results are in accordance with the findings of the current study.

Previous studies regarding accuracy of guided surgery protocols used superimposition of two STL files: first coming from implant planning software and second from post-operative CBCT images. This superimposition was performed using different software such as the Geomagic [12, 21]. However, a second CBCT could increase the amount of distortion due to artifacts and expose the patient to more important irradiation. In a study conducted by Schnutenhaus et al. in 2016, the final implant position was obtained by taking a conventional impression, scanning it, and superimposing the digitalized model with the planned file using Geomagic software [23]. In the current study, the two optical scans of VI and PI were superimposed based on the same matrices containing 3D coordinates of the initial digital scan. This novel superimposition method did not require additional irradiation nor other software which may induce further distortions. No studies comparing these different assessment techniques were found in the literature.

The limitation of our study is with the small sample size in regard with the different implant macro-design and different length.

## Conclusion

CIGS was a reliable choice for optimal prosthetically-driven implant surgery in partially edentulous spaces. However, some steps of CIGS were important sources of inaccuracy such as CBCT artifacts, superimposition technique, implant length, and surgical guide design. While the largest part of distortion originated from the surgical process, CAD/CAM manufacturing process did not result in any distortions. Controlling the distortion parameters decreases the amount of CIGS inaccuracy. Future studies with larger sample sizes must be conducted to validate the results of this study and compare the accuracy of CIGS in different types of edentulism.

### Electronic supplementary material

Below is the link to the electronic supplementary material.

Supplementary Material 1

## Data Availability

The authors confirm that there is no associated data included in this manuscript.

## Abbreviations

CAD-CAM: Computerized aided design-Computerized aided manufacturing
3D: Three dimensional
SIGS: Static Implant guided surgery
CIGS: Computerized Implant Guided Surgery
CBCT: Cone beam computed tomography
ASA: American Society of Anesthesiology score
IRB: Institutional Review Board
CPHS: Committee for the Protection of Human Subjects
DICOM/ DCM: Digital Imaging and Communications in Medicine
STL: Standard Tessellation Language
3oxz: 3Shape Order Exchange Zip File
BL: Bone level implant
BLT: Bone level tapered implant
IT: Insertion torque
RFA: Resonance frequency analysis
ISQ: Implant stability quotient
VI: Virtual implant position
PI: Placed implant position
mm: Millimeter
OD: Origo Displacement
HD: Horizontal Displacement
ED: Error Depth
EA: Error Angle
