# Supplementary material for: Accuracy of computer-guided implant surgery in partially edentulous patients: a prospective observational study
Source: Int J Implant Dent. 2024 Jul 16;10:36. doi: 10.1186/s40729-024-00552-z (PMC11252094; doi:10.1186/s40729-024-00552-z)
Supplement: Supplementary file 1 — Supplementary Material 1 [file 40729_2024_552_MOESM1_ESM.docx]

Rebuttal letter

Dear reviewers;

We deeply thank you for giving us the chance to resubmit our manuscript with specific corrections.

Below are the corresponding changes made:

**Reviewer #1:**

**Reviewer #1: Commented [EC2]: Reviewer #1:**

**I'm not so happy with the answer. In the study, the authors refer to clinical cases. The main message (according to the author's answer) concerns the accuracy of the planning on the models. The accuracy in the clinical situation in patients has not been scientifically investigated. This is confusing. The question is: is this a clinical or in vitro study?**

Author answer:

We are deeply sorry if there was a misunderstanding of your concern and question. Our study is indeed a clinical study where we assessed the accuracy of the CIGS on patient since we compared the final position with the planed position using a special deviation tool (Software designed by 3Shape company) which superimpose the file coming from an intra-oral surface scan using a scan body mounted on the placed implant, with the exported file of the planed implant.

All these details were mentioned on paragraph: “Guided implant surgery protocol “ & paragraph “Measured parameters/ 1.Assessment of the deviations.”

If the word “assessment” in line 149 seems committing us for higher level of scientific assessments, we changed the sentence to “Basically evaluate”

If the way we describe it in our manuscript is not clear enough, would you please advise how would you need us to reformulate it.

**Commented [EC3]: Reviewer #1:**

**- is o.k.**

Author answer:

We thank you.

**Commented [EC4]: Reviewer #1:**

**- please remove this information from the manuscript.**

**It gives the impression that using Amoxi would have an impact on the results. However, this was not examined in this study. And there is no scientific background.**

Author answer:

Information removed.

**Commented [EC5]: Reviewer #1:**

**Ok, then please add 2-3 sentences and describe why you measured ISQ. Please also reference literature.**

Author answer:

The following paragraph was added: “The ISQ records were only scored  to follow with time the evolution of the implant stability, since it is considered an assessment which can provides prospective monitoring and shows fluctuations in stiffness of the implant interface as bone matures from primary to secondary contact. In the current study the ISQ was only a variable used to monitor the behavior of the secondary stability after immediate loading.”

A reference was added as well.

**Commented [EC7]: Reviewer #1:**

**I'm sorry, but it's still not clear to me how and with what (for example: X-ray, measuring probe) the inaccuracy was determined. The statement that clinical testing has been carried out to determine whether the implants are correctly positioned is not scientific. It is not clear from the information how often the splinted pre-milled crowns had errors.**

Author answer:

We apologize and removed the word assessment and replaced by “A preliminary clinical visual check”. Our study is not based at all on the preliminary clinical assessment, it is based on the evaluation after eight weeks where an intra-oral scan was used with a digital scan body on the placed implant…etc, as explained on our above answer to your previous remark: Reviewer #1: Commented [EC2]: Reviewer #1.

Kindly see line 210 till 218.

We also changed some words to make the idea clear. Kindly read again the changes from line 203 till 225 and advise.

Finally, again we appreciate your meticulous review, we hope that this last version will respond to your expectations.

Respectful regards.

Emile Chrabieh
